# Unraveling metabolism underpinning biomass composition shift in *Scenedesmus obliquus* under simulated outdoor conditions using ^13^C-fluxomics

**DOI:** 10.3389/fpls.2025.1637152

**Published:** 2025-12-17

**Authors:** Arnav Deshpande, Bridgie Cawthon, Jessica Loob, Stefanie Van Wychen, Lieve M. L. Laurens

**Affiliations:** 1 BioEconomy and Sustainable Transportation Directorate, National Renewable Energy Laboratory, Golden, CO, United States; 2 Renewable Resources and Enabling Sciences Center, National Renewable Energy Laboratory, Golden, CO, United States

**Keywords:** algae, metabolic flux, biomass composition, simulated raceway, nitrogen depletion, lipids, carbohydrates

## Abstract

To render the resulting biomass more attractive and amenable for utilization as the basis for low-carbon intensity bioproducts, single-celled algae need to be biochemically and metabolically poised to assimilate and store the delivered carbon in the fastest and most efficient manner. Accelerating biochemical carbon storage, as primarily carbohydrates or lipids, is critical to achieve the high carbon capture potential that is assigned to algae. To guide strain optimization and engineering for maximizing carbon capture and storage, it is essential to elucidate the link between carbon metabolism and biomass composition. Most published metabolomics work in algae remains largely restricted to ideal and simplified environmental conditions in model organisms, thereby limiting their translation to outdoor implementation. In this work, we utilize ^13^C isotopic labeling to characterize distinct intracellular metabolic fluxes before, during, and after nitrogen depletion-induced compositional shifts in *Scenedesmus obliquus* UTEX 393. The results indicate that a transition to carbohydrates is characterized by diverting flux to starch instead of replenishing the Calvin cycle for CO_2_ fixation whereas the subsequent transition to lipids is fueled by NADPH produced by upregulating the phosphoenolpyruvate carboxylase (PEPC)–malic enzyme (ME) cycle flux. Our work highlights bottlenecks to carbohydrate- and lipid-rich biomass and can guide implementable strategies to control the fate of fixed carbon in *S. obliquus*.

## 1 Introduction

The production of biofuels and biochemicals in the context of building a strong bioeconomy requires sustainable photosynthetic hosts and scalable farming approaches, for which algae such as *Scenedesmus obliquus* UTEX393 (referred to as UTEX393) are uniquely suited and widely studied (Hannon et al., 2010; Laurens et al., 2017; Feng et al., 2022). Compared to terrestrial plants, algae have higher areal productivity and can be cultivated in aqueous land-based systems on non-arable land, leaving arable land use for food production (Benedetti et al., 2018). However, significant improvements are necessary to reduce cultivation costs and improve biomass compositional quality, e.g., increases storage carbon such as lipid or carbohydrates (Hannon et al., 2010; Benedetti et al., 2018; Klein et al., 2023). The carbohydrate and lipid fraction of biomass are typically enriched during later stages of cultivation when nutrients such as nitrogen and phosphorus are depleted, and the rates at which the compositional shift happens are important determinants of the cells’ physiological capacity. Often the compositional shift reduces instantaneous biomass productivity, and thus, this can lead to higher production costs (Klein et al., 2023). There is thus an urgent need to identify solutions to enable a rapid biomass composition shift from protein to carbohydrate and lipids without sacrificing biomass productivity. This will require the identification of the metabolism underpinning the shift in biomass composition with metabolomics as well as fluxomics approaches that are likely to play a key role (Hendry et al., 2017; Jazmin et al., 2017).

Metabolomic profiling is a powerful tool to understand the carbon flux and elucidate bottlenecks that slow carbon assimilation, storage, and, ultimately, productivity. Metabolomics has been used in algae to study the response of cold stress in *S. obliquus* UTEX393 (referred to as UTEX393) (Calhoun et al., 2021) and salt stress in *Monoraphidium minutum* 26BAM (Calhoun et al., 2022) and UTEX393 (Kruse et al., 2025). Furthermore, the effect of diel cycling has also been investigated (Sarkar et al., 2019; Werner et al., 2019; Jaiswal and Wangikar, 2020). Genome-scale metabolic models have been constructed for model algae such as *Chlamydomonas reinhardtii* (May et al., 2008), and a flux balance analysis (FBA) approach has been utilized with minimization of light uptake, minimization of flux magnitudes, maximization of adenosine triphosphate (ATP) yield, and minimization of ATP demand as objective functions in organisms such as *P. tricornutum* and *C. reinhardtii* (Kroth et al., 2008; Boyle and Morgan, 2009; Kliphuis et al., 2012). However, constraint-based approaches are limited by their reliance on choice of the objective function, which may not truly represent and dictate algal biology in environmental conditions. Instationary metabolic flux analysis (INST-MFA), which uses an isotopic labeling strategy, is an approach that has proven useful in characterizing carbon flux and carbon allocation, or diverting fixed carbon to different biomass components in cyanobacteria and algae (Wu et al., 2015; Yu King Hing et al., 2021; Treves et al., 2022; Jaiswal et al., 2023). However, these studies provide limited information about the metabolic shift to carbohydrates and lipids, are often performed in small-scale shake flasks, and are restricted to growth under nutrient-replete conditions under a laboratory setting, further limiting their translation to outdoor relevant conditions.

**Figure 1:**
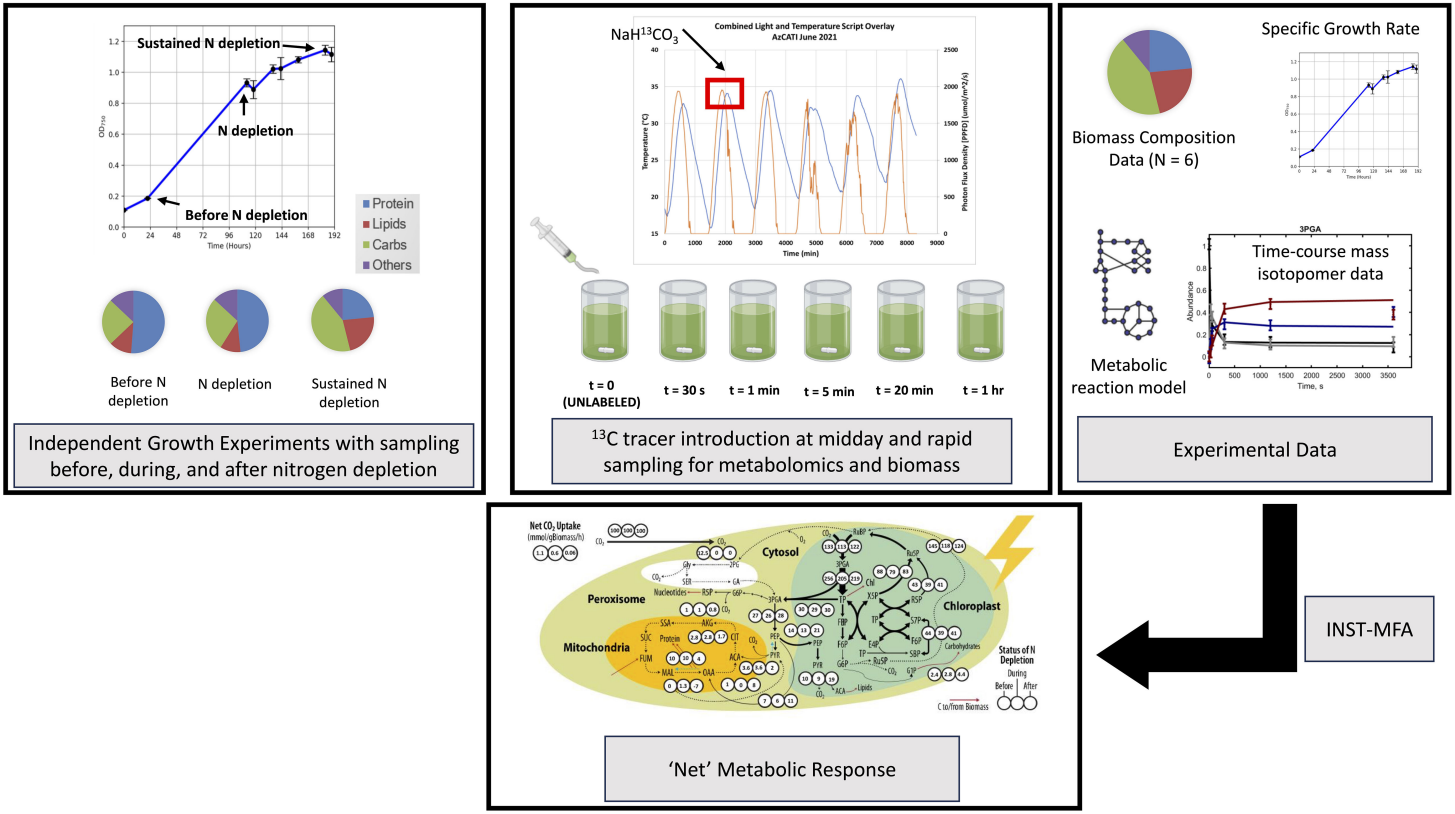
Overview of workflow for quantifying metabolic fluxes under three—before, during, and after sustained nitrogen depletion—conditions that correspond to three varying biomass compositions in UTEX393. Each condition is performed as an independent experiment where ^13^C labeling occurs at midday in 1.5L SAGE bioreactors at pseudo metabolic steady state. Each reactor is treated as a time point for quantifying labeling in metabolites and biomass is collected for compositional analysis. These measurements serve as an input to an INST-MFA model that is used to quantify “net” intracellular fluxes.

To bridge this gap, there is a need to measure metabolic carbon flux in outdoor relevant conditions towards building a framework for identifying strain improvement strategies. Here, we utilize Simulated Algal Growth Environment (SAGE) bioreactors that replicate outdoor light and temperature conditions, and mimic raceway pond biomass productivity and composition to investigate the metabolism underpinning the biomass composition shift in UTEX393. We aim to:

1. Identify and quantify the rates of composition shift dynamics in UTEX393.
2. Develop and utilize a ^13^C isotopic labeling-based fluxomics approach (**Figure 1**) to characterize intracellular metabolic fluxes before, during, and after nitrogen depletion in outdoor relevant UTEX393 to allow us to track metabolism at midday in three distinct nutrient and biomass composition conditions to uncover the metabolism underpinning the shift in composition in outdoor conditions.
3. Investigate if INST-MFA may be utilized to obtain “net/representative” intracellular metabolic fluxes, from isotopic labeling data generated from simulated raceway conditions.
4. Perform preliminary experiments to address bottlenecks from (Feng et al., 2022) and (Laurens et al., 2017) to improve growth and lipid/carbohydrate accumulation.

## 2 Materials and methods

### 2.1 Algae growth conditions

UTEX 393 was routinely cultured and maintained in liquid BG-11 medium consisting of the following components in 1 L of 18.2 MΩ-cm water: 395.12 mg of NH_4_HCO_3_, 71.79 mg of K_2_HPO_4_.3H_2_O, 75 mg of MgSO_4_.7H_2_O, 36 mg of CaCl_2_.2H_2_O, 3 mg of citric acid, 3 mg of ferric ammonium citrate, 40 mg of Na_2_CO_3_, 5.6 g of NaCl, 1 mg of NaEDTA, 2.86 mg of H_3_BO_3_, 1.81 mg of MnCl_2_·4H_2_O, 0.22 mg of ZnSO_4_·7H_2_O, 0.39 mg of Na_2_MoO_4_·2H_2_O, 0.079 mg of CuSO_4_·5H_2_O, and 0.0404 mg of Co(NO_3_)_2_·6H_2_O. The cultures were maintained in a shaker incubator at 130 rpm aerated with 1% CO_2_, 50 µmol m^−2^ s^−1^ light intensity, and set to 27 °C.

For biomass composition shift dynamics experiments, a custom-built 18-position (1-L operating volume) high-throughput bioreactor system (Simulated Algae Growth Environment (SAGE)#5, **Supplementary Photo S1**) was employed. This reactor was programmed to provide constant light from the top at 1,000 µmol m^−2^ s^−1^, temperature was maintained at 25°C, and air enriched with 2% CO_2_ was sparged at 60 mL/min.

To perform experiments in the custom-built SAGE #3 bioreactor (**Supplementary Photo S2**) to simulate raceway pond cultivation, the media was modified to contain 141.11 mg of NH_4_HCO_3_ and 25.9 mg of K_2_HPO_4_.3H_2_O while keeping other components the same to induce nutrient depletion. The cultures in the 6-position 1.5-L SAGE bioreactor were maintained under June 2021 light and temperature profile (**Supplementary Figure S1**) observed at Arizona Centre for Algae Technology and Innovation (AzCATI). The reactor bottles were shaded such that light was available only from the top. The reactor was aerated with 40 mL/min air and operated under pH feedback control by injecting CO_2_ using sparge stones at 5 mL/min to maintain pH setpoint at 7.5 during daytime. Growth was routinely monitored by tracking optical density at 750 nm (OD_750_) using a spectrophotometer.

### 2.2 Transient ^13^C isotopic labeling experiment

Transient ^13^C labeling was performed at midday on days 2, 4, and 9 to represent states before, during, and after nitrogen depletion conditions in the SAGE #3 bioreactor system. Each experiment (before, during, and after) was performed separately due to the destructive nature of sampling. The entire labeling experiment occurred from 30 min before the midday light peak to 30 min after.

CO_2_ supply via pH feedback was stopped 5 min before ^13^C label introduction via a bolus addition of 11.76 mM NaH^13^CO_3_ (99%) (Cambridge Isotope Laboratories, Andover, MA) and 5 mM N-2-hydroxyethylpiperazine-N’-2-ethanesulfonic acid (HEPES) buffer (Sigma-Aldrich, St. Louis) to each reactor. NaH^13^CO_3_ was preferred to ^13^CO_2_ to avoid gas–liquid mass transfer limitations and achieve rapid equilibrium. The buffer is needed to maintain culture pH at ~7.5. Six time points were chosen for metabolite sampling: 0 (unlabeled), 30 s, 1 min, 5 min, 20 min, and 1 h, and each of the six reactors represented a separate time point. These time points were chosen to capture the complete labeling dynamics of the rapidly labeling Calvin Benson Bassham (CBB) cycle metabolites as well as relatively slower labeling metabolites. Depending on culture density, 60–300 mL of culture was sampled using a 300-mL syringe, rapidly filtered via a fast filtration setup using 9-cm Fisherbrand glass fiber filters (catalog no. 09-804-90A) and quenched in liquid nitrogen bath. The quenching method followed a recommended approach for suspension cell cultures, based on rapid filtration, followed by quenching in liquid nitrogen prior to cold methanol extraction, minimizing metabolites losses (Sake et al., 2020; Jaiswal et al., 2022; Wang and Young, 2022). Sample collection through to quenching in liquid nitrogen consistently took up to 15 s and was performed by a team of two researchers. The remaining culture was used for analysis of biomass composition, total nitrogen, and ash-free dry weight (AFDW) in accordance with NREL laboratory analytical procedures (Van Wychen et al., 2021).

### 2.3 Metabolite extraction and analysis

Biomass filters stored at −80 °C were kept on dry ice prior to metabolite extraction. Filters were then transferred to 15-mL centrifuge tubes on dry ice and extraction was performed by adding 2 mL of methanol at −20 °C followed by maceration until a pulp was formed. Subsequently, 2 mL of chloroform at 4 °C was added to the macerated pulp. Once extraction was complete with the methanol:chloroform mixture, 5 mL of chloroform and 1 mL of 0.05% ammonium hydroxide (pH ~10.4) were added for phase separation. The extracts were vortexed gently followed by centrifugation at 4 °C at 2,500 *g*. The clear layer on top was removed, filtered using a syringe filter, diluted in acetonitrile (ACN) (3:1 ACN: extract), and finally transferred to a vial prior to injection.

Chromatographic separation was achieved using hydrophilic interaction chromatography (HILIC) using a BEH Amide column (1.7 µm, 2.1 mm × 150 mm, ACQUITY Premier BEH Amide, Waters Corporation) by a gradient method using a Thermo VF-P20-A pump system as previously described (Steichen et al., 2024). Solvent A: 20 mM ammonium acetate, 15 mM ammonium hydroxide in 97% 18.2 mΩ-cm water, and 3% acetonitrile; solvent B: 20 mM ammonium acetate, 15 mM ammonium hydroxide in 95% acetonitrile, and 5% 18.2 mΩ-cm water were used in the gradient method as follows: 90% solvent B for 1 min followed by a linear gradient down to 75% solvent B for 23 min, a linear gradient down to 45% solvent B in 2 min, a linear gradient down to 25% solvent B in 4 min, followed by a step to the starting composition of 90% solvent B, and hold for 6 min for column regeneration for a total run time of 36 min. The flow rate was kept constant at 0.2 mL/min, and column temperature was maintained at 25 °C. Mass spectrometer data were collected using a Thermo Scientific Q-Exactive mass spectrometer in negative ion mode. The scan range was from 60 to 900 *m*/*z* with a resolution of 140,000. Data were processed using TraceFinder 5.1 and analyzed using Rstudio 4.2.0. Experimental data files were uploaded to Metabolomics Workbench and are available under Study ID ST003583 (Sud et al., 2016).

### 2.4 INST-MFA

Intracellular metabolic fluxes were quantified using INCA (Rahim et al., 2022). A compartmentalized metabolic reaction network, previously used for *P. celery*, included the Calvin cycle, oxidative pentose phosphate pathway, glycolysis, transport reactions, tricarboxylic acid (TCA) cycle, and biomass sinks (Steichen et al., 2024). It was assumed that there was 3% residual ^12^CO_2_ after the pulse of saturated ^13^C-bicarbonate addition as previously described (Abernathy et al., 2017). Dilution reactions were added to account for inactive pools and metabolite channeling (Yu King Hing et al., 2021). Dilution pools were also required to achieve acceptable fits based on chi-square test, which can be attributed to the “real world” or unideal conditions in the photobioreactors. Biomass production was modeled using reactions for lipid, chlorophyll, protein, carbohydrate, and nucleotide synthesis. The protein biosynthesis equation was developed based on the amino acid composition of protein of UTEX393. Detailed information on biomass synthesis equations is provided in **Supplementary Table 2**. Best-fit estimates for each strain were computed with at least 50 repetitions with different random initial guesses to obtain a fit. Detailed information about the model and fits is available in **Supplementary Table 3**.

### 2.5 Malic acid supplementation experiments

For performing malic acid supplementation experiments in UTEX 393 cultures, the media was modified to contain 141.11 mg of NH_4_HCO_3_, 25.9 mg of K_2_HPO_4_.3H_2_O, 4.77 g of HEPES buffer, and 10 mM of malic acid, while keeping the other components the same. Media pH was adjusted to 7.5 using NaOH. Spectinomycin (25 µg/mL) was added in shake flask scale experiments. For shake flask scale experiments, triplicate 100-mL cultures in the buffered BG-11 media with and without supplemented malic acid were maintained in a shaker incubator at 130 rpm aerated with 1% CO_2_, 50 µmol m^−2^ s^−1^ light intensity, and set to 27 °C. Growth was routinely monitored by tracking optical density at 750 nm (OD_750_) using a spectrophotometer, and 50-mL aliquots were taken on days 4 and 10; replicates were pooled and freeze dried and analyzed for fatty acid methyl ester (FAME) and carbohydrate content. For SAGE #5 experiments, 600-mL cultures in biological triplicates were tested with or without malic acid (10 mM) in buffered BG-11 medium. Reactors were under an environmental simulation representative of Fall 2019 at AzCATI (**Supplementary Figure S2**) with continuous 1% CO_2_ sparging at 40 mL/min. Biomass growth was monitored using ash-free dry weight measurements and 100 mL was harvested on days 5 and 8 for analysis of ash-free carbohydrate and ash-free FAME content.

## 3 Results and discussion

### 3.1 UTEX 393 is biochemically poised for carbohydrate and subsequent lipid accumulation

Some strains, like *S. obliquus* UTEX393, are biochemically poised to accumulate carbohydrates first and subsequently accumulate lipids on continued growth under nitrogen depletion. We first sought to uncover time-dependent biomass composition shift dynamics of UTEX 393 as nitrogen depletes. To test this, we utilized SAGE#5, an 18-position high-throughput bioreactor system. Twelve cultures were grown under constant light from the top at 1,000 µmol m^−2^ s^−1^, temperature was maintained at 25 °C, and air enriched with 2% CO_2_ was sparged at 60 mL/min. Three replicate reactors were harvested on days 1, 4, 5, and 6 for biomass composition. The biomass composition data are shown in **Table 1** and the growth data (AFDW) are shown in **Supplementary Figure S3**.

**Table 1:** Time series biomass composition of UTEX 393. Data represent mean ± standard deviation of three biological replicates.

Component	Day 1	Day 4	Day 5	Day 6
Ash	$	3.70 ± 0.08	3.23 ± 0.07	3.09 ± 0.07
FAME	13.37 ± 0.41	22.53 ± 0.53	27.88 ± 1.40	34.11 ± 1.70
Protein^*^	34.75 ± 5.26	12.28 ± 0.3	11.03 ± 0.66	9.88 ± 0.33
Carbohydrates^**^	21.06 ± 0.37	47.82 ± 0.59	44.05 ± 0.46	39.20 ± 2.21

$ Not enough biomass available for measurement.

*Protein assumes an N conversion factor of 4.78.

**Carbs contain neutral sugars, galactosamine, glucosamine, and mannitol.

UTEX 393 biomass composition dynamics reveal that under nitrogen depletion, newly fixed carbon flux is first diverted away from predominantly protein biosynthesis to carbohydrates, which account for nearly 48% of the biomass on day 4. This is subsequently followed by lipid biosynthesis, which increases from 22% to 34% from day 4 to day 6, while carbohydrates drop from 48% to 39%. The AFDW and composition data together do not indicate the catabolism of either proteins or carbohydrates into lipids as we do not see any decrease in total amount of any biomass component with time (**Supplementary Figure S4**).

These results highlight important carbon flux dynamics to major biomass sinks but do not shed light on the underlying intracellular fluxes and mechanisms that position UTEX 393 to first accumulate carbohydrates and then lipids.

### 3.2 ^13^C labeling dynamics reveal the metabolism underpinning the composition shift in raceway conditions

Uncovering metabolic changes that occur as nutrients deplete and composition starts to shift away from primarily protein is essential to develop strategies to direct fixed carbon to valuable fractions such as lipids, thereby increasing the value of biomass and algae bioeconomy. To study biomass composition shift in the most relevant context, we cultured UTEX 393 in SAGE#3, a reactor that closely mimics raceway pond conditions and operation by controlling light (incident from the top) and temperature cycles, and pH feedback-based CO_2_ supply. Mixing parameters of the reactor have been tuned to mimic outdoor biomass productivity and nitrogen depletion rates. SAGE#3 (**Supplementary Photo S2**) represents the model system used to investigate the metabolism that governs composition shift dynamics in outdoor environments.

**Figure 2:**
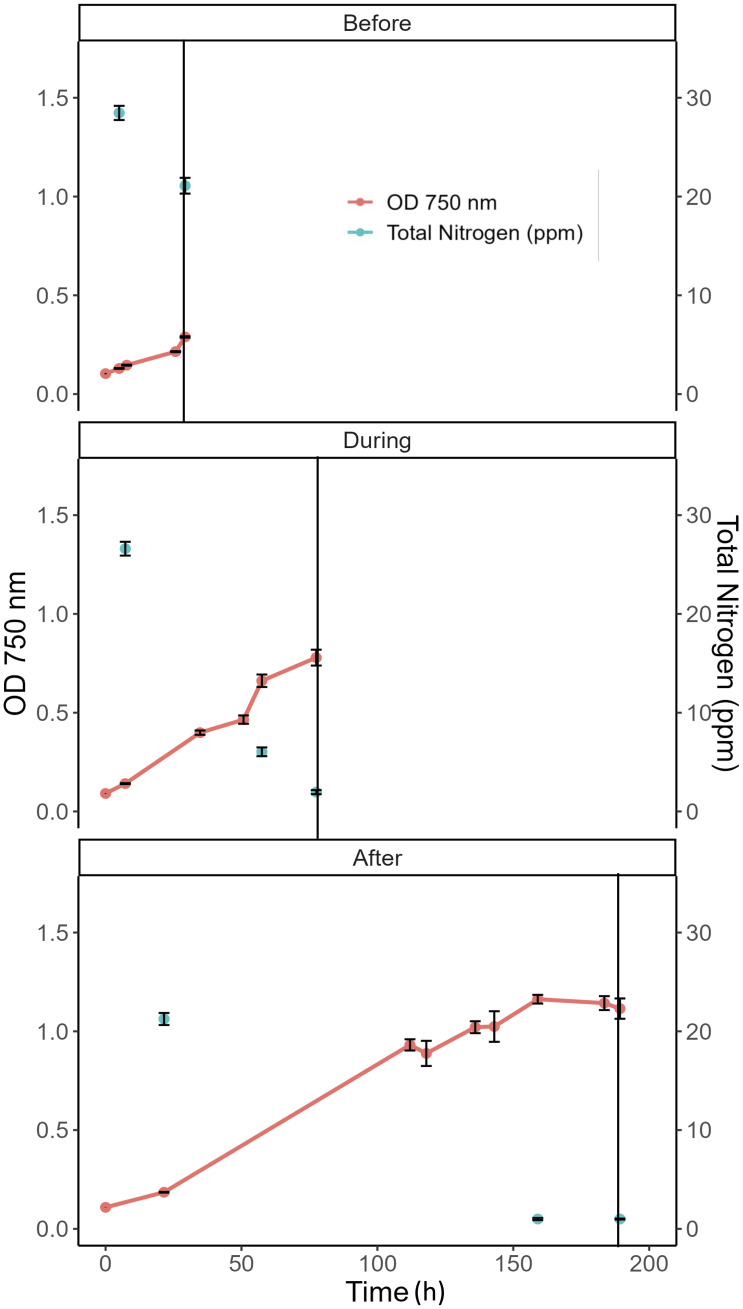
Growth (OD_750_) and total media nitrogen (ppm) for three separate experiments used for ^13^C labeling indicated by the vertical solid black line for the three stages: “Before”, “During”, and “After”. Data are shown as the mean and standard deviation of six biological replicates.

Preliminary experiments were conducted in SAGE#3 to determine growth curves, total media nitrogen depletion rates, and biomass composition shift dynamics under raceway pond conditions to carefully design a ^13^C labeling experiment (**Figure 1**). We sought to introduce ^13^C label to trace carbon flow in three distinct stages: “Before”, “During”, and “After” media nitrogen depletion. The “Before” state represents actively growing cells at lower cell density with sufficient nitrogen available in the media; “During” represents the state where total media nitrogen is just depleted, and elicits a metabolic response that marks the beginning of composition shift; while the “After” state represents the state when cells continue in culture under nitrogen deprivation stress at a higher cell density for a continued period. **Figure 2** shows the growth curves and total media nitrogen with solid black lines indicating time of labeling. The media total nitrogen data indicated that we were able to accurately perform labeling for the “During” stage, as media nitrogen had just depleted.

Biomass was harvested at the end of the labeling experiment, and the data are shown in **Table 2**. We find that under diel raceway operation conditions, the composition shift dynamics are similar to that under constant light. An increase in carbohydrates is initiated just as nitrogen is depleted with an increase from 20% to 23%. Continued growth under nitrogen depletion results in carbohydrates accumulating to more than 40% while there is a nearly threefold increase in lipids from 8% to 22%.

**Table 2:** Biomass composition of UTEX 393 under raceway conditions harvested before, during, and after media nitrogen depletion.

Component	Before	During	After
Ash	5.96 ± 0.12	5.04 ± 0.15	2.99 ± 0.13
FAME	9.67 ± 0.18	8.65 ± 0.09	22.13 ± 0.59
Protein^*^	42.33 ± 0.6	39.47 ± 0.41	22.96 ± 1.25
Carbohydrates^**^	20 ± 0.65	22.75 ± 0.91	42.04 ± 0.39

*Protein assumes an N conversion factor of 4.78.

**Carbs contain neutral sugars, galactosamine, glucosamine, and mannitol.

Table legend: Data represent mean and standard deviation of six biological replicates. Detailed composition data are provided in the **Biomass Composition Supplementary File**.

Under a diel cycle with continuously changing light and temperature, metabolic flux is dynamic and carbon fixation and allocation is closely tied to light and temperature. Therefore, under all the three stages, we chose midday, where light and temperature peak for ~2 h and carbon fixation is the fastest. For each stage, ^13^C labeling was initiated 30 min before midday peak intensity and the last sample was collected 30 min after midday peak light intensity. The light and temperature profiles are shown in **Supplementary Figure S1**. This strategy enabled the whole labeling experiment to be completed with light and temperature variations less than 100 µmol m^−2^ s^−1^ and 3 °C respectively.

The labeling dynamics of several metabolites under the three stages are shown in **Supplementary Figure S5**. We find metabolites that label the quickest before nitrogen depletion while the slowest label incorporation is observed in the after stage. This is expected as the specific growth rate (**Supplementary Table S1**) is the highest in the before stage and the slowest in the after stage. However, the labeling data must be normalized to the different rate of carbon uptake to understand differences in carbon partitioning at important intracellular nodes. To do this, we fit the time course total ^13^C incorporation data to logistic equation as previously described (Caroli and Frisoni, 2010; Huß and Nikoloski, 2023):


$$Fraction\;13C=\frac{Asym}{1+\;e^{\frac{xmid-t}{scal}}}$$

Here, the *Asym* parameter represents the maximum label incorporation; *xmid* represents the time required to label 50% of the maximum label incorporation, while *scal* is indicative of the maximal rate of label incorporation. This allows us to compare *xmid*, which indicates the time required to label 50% of the maximum pool labeling. Fits were obtained using RStudio 4.2.0 using the SSlogis model. To normalize for the difference in growth rates in the three states, we chose to normalize *xmid* to *xmid_3PGA_*, since 3-phosphoglycerate (3PGA) is the first metabolite produced by the RuBisCO carboxylation reaction. This renders the labeling of all metabolites downstream comparable between the three stages with higher values of *xmid* indicating lower flux.

**Figure 3:**
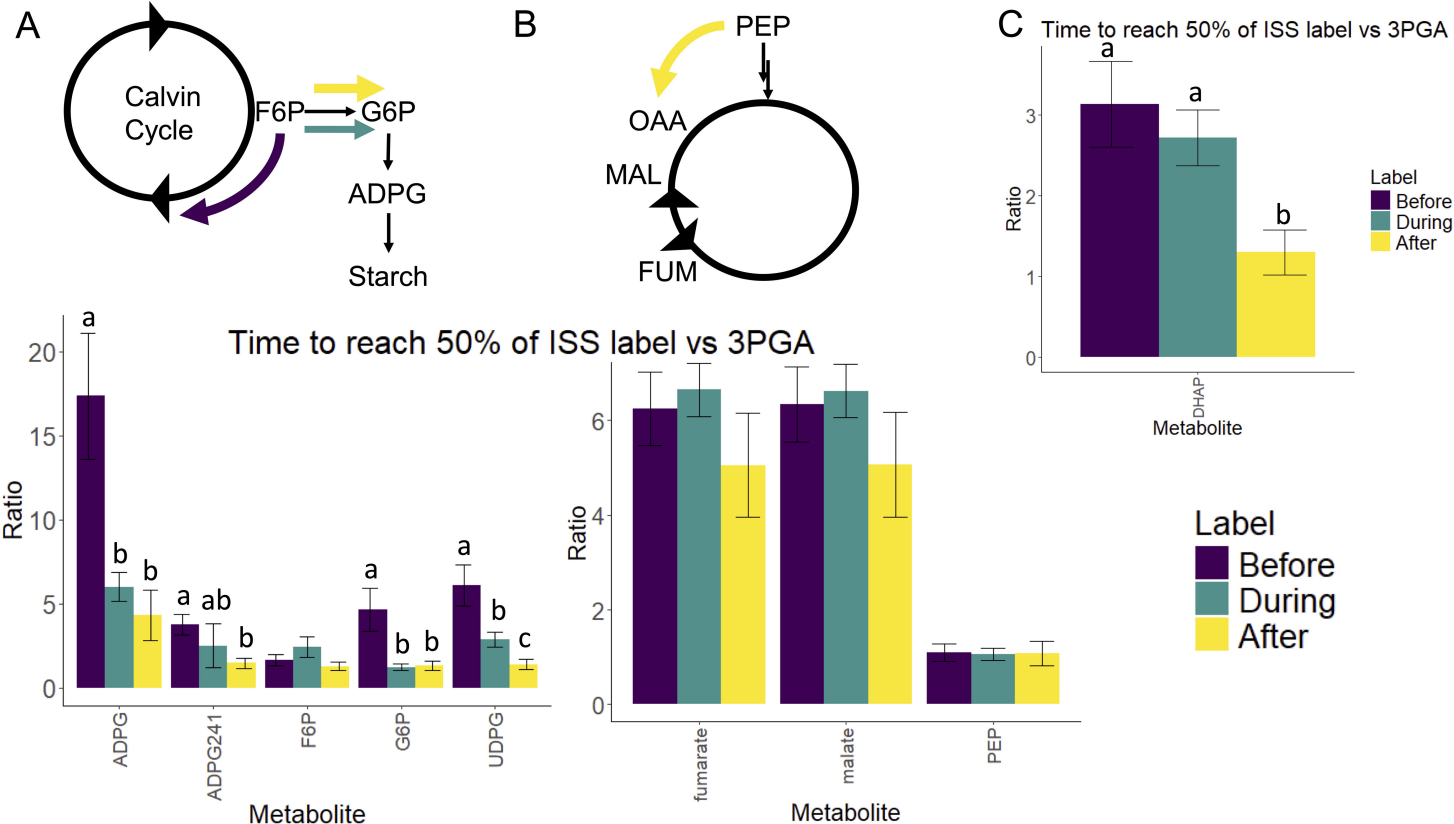
Pictorial representation showing carbon partitioning at **(A)** F6P node and **(B)** PEP mode accompanied bar graphs showing the ratio of time to reach 50% of maximum ^13^C label to that of 3PGA. **(C)** Time to reach 50% of maximum ^13^C label in DHAP to that of 3PGA. Data shown are calculated as *Ratio = xmid_metabolite_/xmid_3PGA_*, where the *xmid* parameter is calculated from logistic fit as described in this section. Error bars are calculated from the standard error of the *xmid* parameter from logistic fit. Letters are used to denote significant differences (*p* < 0.05) based on Wald’s *Z* test. ADPG, adenosine diphosphate glucose; UDPG, uridine diphosphate glucose; DHAP, dihydroxyacetone phosphate; PEP, phosphoenolpyruvate; F6P, fructose 6-phosphate; G6P, glucose 6-phosphate.

We find clear differences in carbon partitioning at F6P and PEP nodes as shown in **Figure 3**, and that it takes a similar amount of time to label F6P under all three stages; however, downstream metabolites in the starch biosynthesis pathway such as G6P and ADPG (ADPG241, which represents the 6-carbon fragment of ADPG derived from G6P) take a much shorter time to label under continued nitrogen depletion “after” stage when compared to the nitrogen-replete before stage as shown in **Figure 3A**. G6P labels nearly 3.5-fold faster after nitrogen is depleted (*p* = 0.011 Before–During, and *p* = 0.009 Before–After), when compared to the nitrogen-replete condition. This indicates that under nitrogen-replete conditions, carbon is shuttled back to the Calvin cycle for regeneration of CBB precursors to fix new carbon, whereas, under nitrogen-deplete conditions, carbon is driven to G6P branch for starch biosynthesis. This begins starch accumulation, as well as may be associated with reduced carbon fixation due to limited ribulose 1,6-bisphosphate (RuBP).

We also find differences in carbon portioning at the PEP node as shown in **Figure 3B**. PEP labels rapidly in all three conditions. PEP carboxylase can drive carbon flux into the TCA cycle by catalyzing the conversion of PEP to oxaloacetate (OAA). OAA can be converted to malate and fumarate through the reversible activity of malate dehydrogenase (MDH) and fumarase. We see that although PEP labels at a similar rate, malate and fumarate label ~30% quicker under continued nitrogen depletion in the after stage when compared to the nitrogen-replete stage. Phosphoenolpyruvate carboxylase (PEPC) also results in the capture of a CO_2_ molecule, which may be of greater importance when Calvin cycle regeneration flux is lower under the nitrogen-depleted scenario as shown in **Figure 3A**. We also find that DHAP, a precursor for phospholipid biosynthesis, labels nearly 2.5- and 2-fold faster in the after stage compared to before (*p* = 0.0022) and during (*p* = 0.0014) stages, respectively, indicating that it has a higher flux after nitrogen is depleted, which may be implicated in greater phospholipid biosynthesis (**Figure 3C**).

### 3.3 Instationary metabolic flux analysis reveals a PEPC–ME cycle that drives lipid accumulation

To further understand intracellular flux distributions under these three stages of nitrogen availability, we tested if we could utilize the INST-MFA approach. This approach relies on several assumptions that include metabolic steady state during labeling (metabolite pools and fluxes are constant), as well as a homogeneous cell population. Cultures in raceway conditions are unlikely to meet these criteria since metabolism is constantly adapting to external light and temperature stimuli. Furthermore, as cells reach a higher density, cell subpopulations with altered metabolism may exist due to cell shading effects. Therefore, our goal was to test if we could use the INST-MFA approach to uncover the “net” or “effective” metabolic response of the cell population. To minimize the change in external conditions, we restricted the sampling to be conducted in the peak light and temperature hours where light and temperature variations were less than 100 µmol m^−2^ s^−1^ and 3 °C. To address the issue of cell subpopulations, we decided to handle that by utilizing dilution pools for 14 metabolites (**Supplementary Table 3, INCA reaction model**). Dilution reactions are required to describe the lack of equilibrium between labeled and unlabeled pools or labeling dilution from unknown resources (Abernathy et al., 2017). Additionally, as in our case, dilution pools may also account for differences in labeling due to cell subpopulations that may be shaded and contribute to higher unlabeled pools. The minimum standard deviation for mass isotopomer data needed to obtain statistically acceptable fits for was 0.045, 0.05, and 0.04 for the conditions “Before”, “During”, and “After” stages, respectively. These values are in a similar range (0.02–0.05) to that used previously in literature (Abernathy et al., 2017; Nakajima et al., 2017; Yu King Hing et al., 2019; Yu King Hing et al., 2021), which were cultured in “ideal” conditions. Sum squared residuals (SSRs) were evaluated between model fits and experimental data. Before: SSR = 261.9 [242.9, 336.9]; During: SSR = 332.2 [242.9, 336.9], and After: SSR = 230.9 [190.8, 275]; all were statistically acceptable by the chi-square test. The value of SSR for all the models was in the acceptable range, indicating that the model fit is suitable. To assess if specific reaction fluxes are different between the models, we can compare if there is an overlap between the 95% confidence intervals between the three different cases (Refer **Supplementary Table 3, Absolute and Normalized Flux Tab**). The experimental and fitted mass isotopomer distributions are shown in **Supplementary Figures S6**-**S8**.

**Figure 4:**
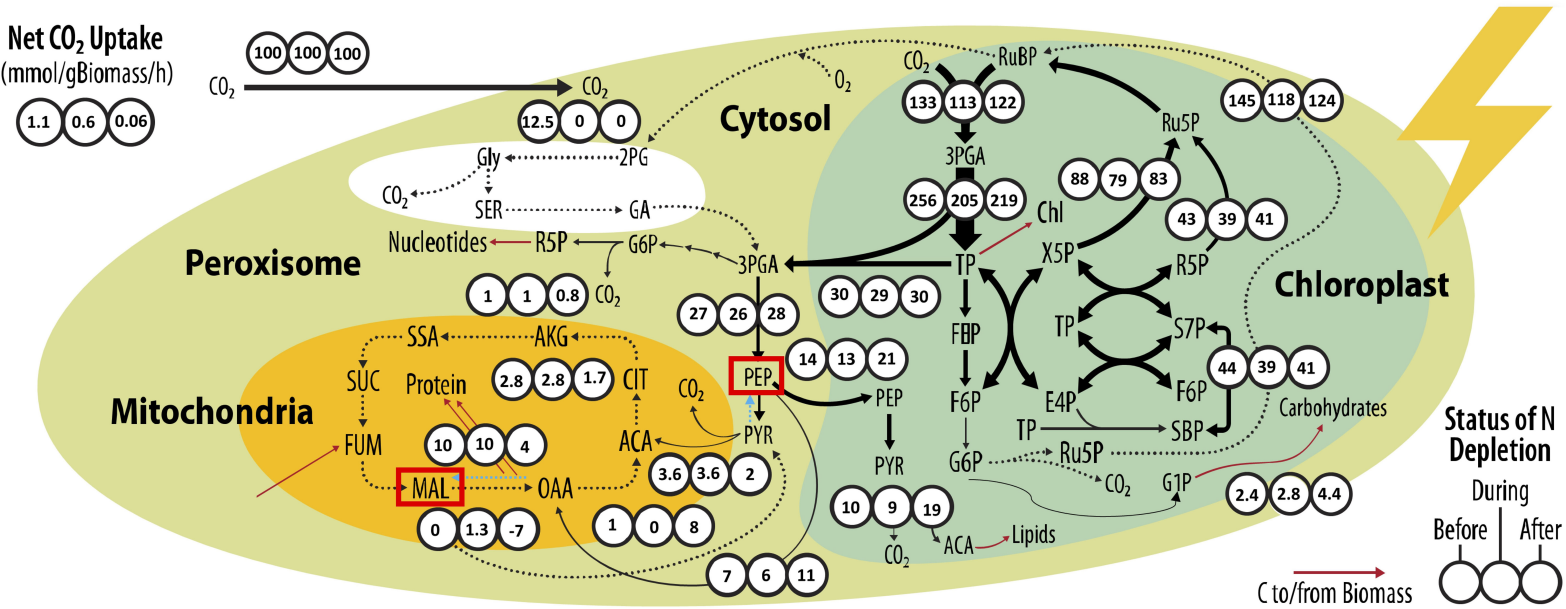
Flux maps determined “Before”, “During”, and “After” nitrogen depletion under raceway pond operation conditions. For each reaction shown, the net fluxes are normalized to 100 units of CO_2_ taken up. All flux values and confidence intervals are given in the MFA **Supplementary File**. Estimated net CO_2_ uptake rates for “Before”, “During”, and “After” stages are 1.1, 0.6, and 0.06 mmol/gBiomass/h, respectively. Abbreviations: ribulose 1,5-bisphosphate (RuBP), 3-phosphoglycerate (3PGA), triose phosphate (TP), fructose 1,6-bisphosphate (FBP), fructose 6-phosphate (F6P), glucose 6-phosphate (G6P), glucose 1-phosphate (G1P), ribulose 5-phosphate (Ru5P), erythrose 4-phosphate (E4P), ribose 5-phosphate (R5P), xylulose 5-phosphate (X5P), sedoheptulose-7-phosphate (S7P), sedoheptulose-1,7-bisphosphate (SBP), phosphoenolpyruvate (PEP), acetyl-CoA (ACA), pyruvate (PYR), glycine (Gly), serine (SER), 2-phosphoglycolate (2PG), glycolic acid (GA), citrate (CIT), alpha-ketoglutarate (AKG), succinic semialdehyde (SSA), succinate (SUC), fumarate (FUM), malate (MAL), and oxaloacetate (OAA). Black arrows represent intracellular flux while red arrows represent fluxes to/from biomass sink. The width of the black arrows is indicative of the magnitude of the flux. The blue arrows highlight reactions that drive flux in the opposite direction in the “After” stage. Red boxes indicate the MAL and PEP node.

The net CO_2_ uptake rate under the three conditions before, during, and after is 1.1, 0.6, and 0.06 mmol/g biomass/h, respectively. As cells grow and culture density increases, cells are subject to self-shading, in addition to depletion in nitrogen availability per cell. Carbon uptake and its link to nitrogen availability has been well studied, showing that nitrogen limitation negatively regulates photosynthesis and carbon assimilation (Turpin, 1991; Zhao et al., 2017; Cointet et al., 2019). To account for these differences in growth, we normalized the uptake of CO_2_ to 100 units in all three stages as shown in **Figure 4**.

INST-MFA reveals several interesting differences between the three stages. Firstly, in the Calvin cycle, we see that the RuBP regeneration is 24% (145 vs. 117) and 17% (145 vs. 124) higher in the cell population in the before stage, when compared to during and after stages. This results in higher RuBisCO carboxylase flux in the before stage. Interestingly, we see that 9% of regenerated RuBP from the Calvin cycle is directed towards photorespiration in the before stage, which is only 3.5% and 2% in the during and after stages, respectively. This indicates that RuBisCO carboxylase activity may be insufficient to handle the higher RuBP regeneration in the before stage. In the during and after stage, RuBisCO carboxylase activity is sufficient to prevent any photorespiration. Previous work has indicated that photorespiration occurs under high light stress conditions (Sunil et al., 2019; Yu King Hing et al., 2021). This may be the case in our system as light penetration is higher in the before stage at lower culture density, as opposed to during and after stages where cell shading occurs. Furthermore, we identify a higher oxidative pentose phosphate flux in the before stage. This pathway yields NADPH and may be activated to counter reactive oxygen species. Previously, this pathway has been upregulated under high light conditions in algae (Steichen et al., 2024) and may indicate that higher light penetration under the low-density before condition is responsible.

Differences in biomass composition necessitates distinct demand for precursors (**Table 2**). A higher protein content derives several precursors from the TCA cycle in mitochondria whereas the production of starch in the chloroplast requires flux derived from G6P. Furthermore, lipid production occurs in the chloroplast with PEP imported from cytosol. This makes the transport between the compartments closely tied to biomass composition. As carbohydrate content increases from 20% to 42% from the before to after stage, we find a nearly twofold flux from G6P to carbohydrates as expected. Interestingly, we find that this does not affect the export of triose phosphates into the cytosol as shown in **Figure 4**. Under continued nitrogen depletion for several days (after stage), there is no longer enough intracellular nitrogen reserve for amino acid biosynthesis, which can be seen by a 45% reduction in flux to TCA cycle via pyruvate dehydrogenase reaction. Instead, we find evidence for a PEP carboxylase–malic enzyme (ME) cycle. Carbon flux from PEP is driven to OAA via the activity of PEP carboxylase that is nearly 50% higher in the after stage compared to the before stage. The reversible action of MDH can convert OAA to malate, which is converted to pyruvate by potentially the NADP-dependent ME. The activity of ME as the primary mechanism to oxidize malate was previously described in cyanobacteria (Katayama et al., 2022). This results in the net production of NADPH via the ME reaction and may then be used to drive lipid biosynthesis. This cycle bypasses pyruvate kinase and pyruvate dehydrogenase, which is the primary mechanism to drive flux to the TCA cycle in the before and during states. The recycled pyruvate is then converted to PEP by pyruvate phosphate dikinase and transported back to the chloroplast for lipid biosynthesis. There is previous evidence of ME involved in contributing to greater lipid content and its downregulation leading to a reduction in algae that provides support to this proposed mechanism (Xue et al., 2016; Yan et al., 2019; Jeon et al., 2021). Fluxes from INST-MFA can be used to calculate the ATP and NADPH demands to inform us about the cell energetic state (**Supplementary Table 3, Co-Factor Demands**). ATP/NADPH demands have an important impact on metabolism by altering linear and cyclic electron flows (Deshpande et al., 2025). Based on the reactions modeled, we find that the ATP/NADPH demand decreases from 1.55 in the before stage, to 1.38 and 1.31 in the during and after stages, respectively. A lipid-rich biomass requires larger amounts of NADPH for biomass production, leading to lower ATP/NADPH demand. Interestingly, just as nitrogen is depleted in the during stage, we see a drop in the ATP/NADPH ratio from 1.55 to 1.38, although lipid composition of biomass is unchanged compared to the nitrogen-replete stage. This indicates that lipid accumulation may be triggered by first switching metabolism to reduce the ATP/NADPH ratio.

### 3.4 Malic acid supplementation enhances lipid accumulation in UTEX 393

Based on the insights from metabolic fluxes at midday over the course of natural nitrogen depletion, we hypothesized that supplementation of malic acid can drive UTEX 393 to preferentially accumulate lipids under nitrogen depletion by boosting ME flux and resulting NADPH production for lipid biosynthesis. We tested this hypothesis by supplementing the media with 10 mM malic acid and tested the effect on growth and biomass composition under continuous light conditions in shake flask cultures as well as under simulated environmental conditions in SAGE#5. Exogenous addition of malic acid represents a quick, indirect test to probe the role of ME activity on biomass composition.

**Figure 5:**
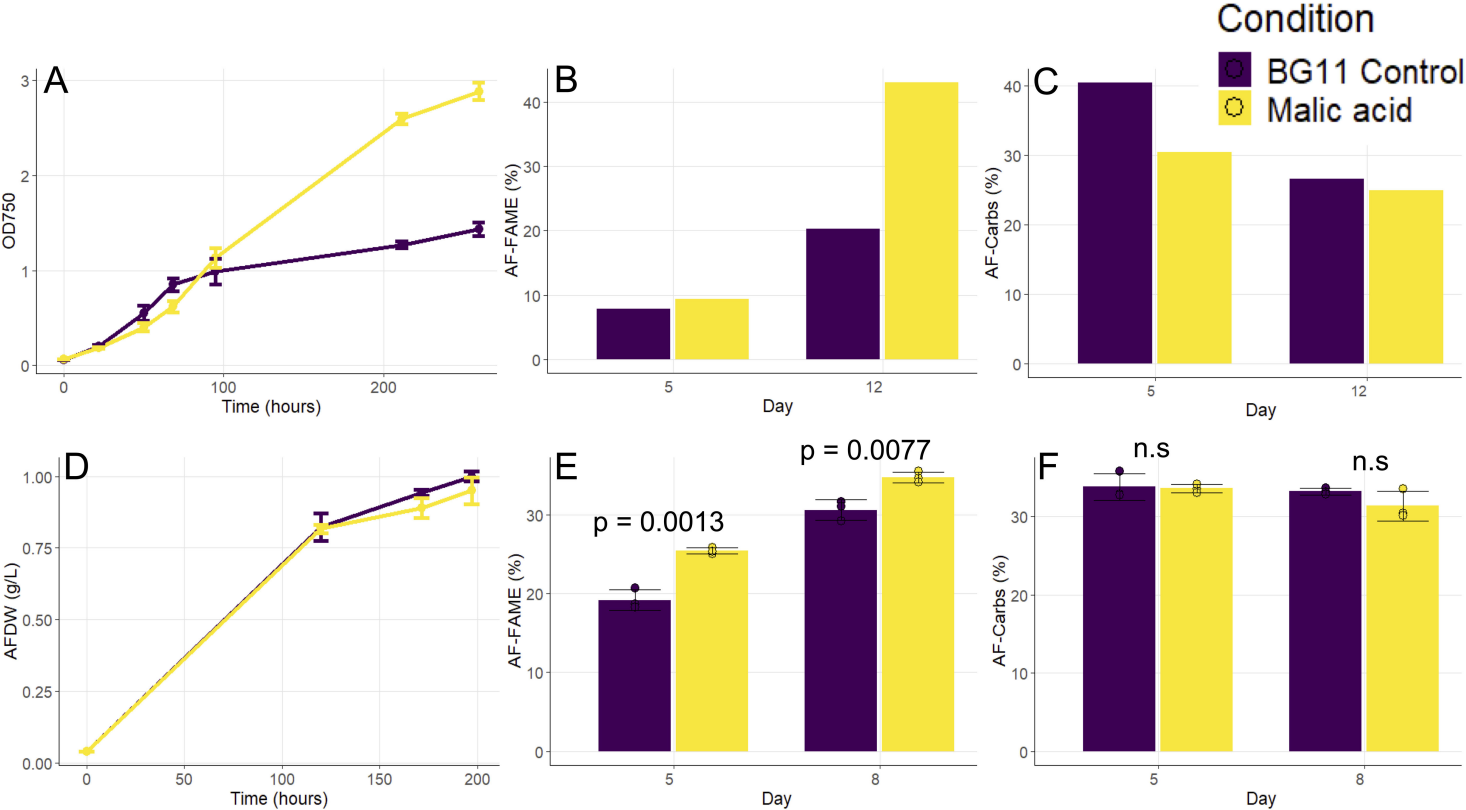
Effect of malic acid supplementation. Growth **(A, D)**, ash-free carbohydrate content **(B, E)**, and ash-free FAME content **(C, F)** under continuous illumination in shake flask cultures **(A–C)** and simulated environmental conditions **(D–F)**. Data represent mean and SD of three biological replicates, except **(B, C)** where data are from pooled biological replicates.

Under continuous illumination in shake flask cultures, we found that malic acid supplementation increased growth rate exceeding that of the control culture (**Figure 5A**). Biomass composition on day 12 showed a remarkable twofold increase in lipid accumulation relative to the control, while the carbohydrate content was similar (**Figures 5B, C**). When cultivated in diel environmental conditions, we found no significant difference in biomass accumulation (**Figure 5D**). The lipid content showed significant improvement on both day 5 and day 8, with nearly 33% and 14% higher accumulation compared to the control (**Figure 5E**). The carbohydrate content showed no change (**Figure 5F**).

These data provide preliminary support that ME flux and NADPH thus produced may limit lipid accumulation in UTEX 393. The flux maps developed in this work represent flux at midday under the three states of nitrogen depletion, but it is unclear what the impact of nitrogen depletion is on metabolism during other parts of the diel cycle.

These data also show that the nearly twofold improved lipid content can be achieved, but it relies on continuous illumination, which is not realistic for large-scale production, with the observed effects diminished under diel environmental conditions. Our work highlights that developing a detailed understanding of carbon flux allocation during physiological changes, thus inducing compositional shifts of the biomass, is useful to identify metabolites (e.g., malic acid) that exhibit a significant impact on the rate of compositional shifts and lipid accumulation. However, attaining sustained improved lipid productivity will require additional efforts beyond malic acid supplementation. Future work is necessary to test whether genetically engineering algae with increased ME flux can deliver the same lipid productivity improvement, or whether other reaction fluxes that increase NADPH availability for lipid biosynthesis are alternative genetic targets. It will be critical to demonstrate the impact of these engineering efforts in challenging real-world diel environments.

## 4 Conclusions

This work presents a detailed study of metabolic carbon flux in diel, environmentally simulated cultivation conditions of a microalgae strain known to exhibit a sequential carbohydrate and lipid accumulation pattern, during three distinct, sequential, physiological phases of cultivation representing three compositional profiles.

This ^13^C labeling study was designed to unravel the metabolic changes underpinning the compositional transition from high protein content biomass under a nitrogen-replete state to a carbohydrate- and lipid-rich biomass under a nitrogen-depleted state in *Scenedesmus* UTEX 393. Our work provides unique insights into algal metabolism as we perform this study in a custom-built reactor equipped to match raceway pond operation. This is crucial to bridge the gap between “ideal” laboratory conditions and “real-world” cultivation conditions.

We show that the transition to high-carbohydrate biomass is characterized by increased flux diverted to starch instead of replenishing the CBB cycle for CO_2_ fixation whereas the subsequent transition to high-lipid biomass is fueled by NADPH produced by the PEP carboxylase–ME cycle. The transition to lipid-rich biomass is activated by first lowering the ATP/NADPH ratio. To test this insight from the ^13^C labeling experiment, exogenous addition of malic acid was performed that increased lipid content by almost twofold, by potentially overcoming the NADPH demand of the lipid synthesis pathway by driving higher NADP-dependent ME flux, highlighting the value in identifying these central metabolic decision nodes. Further model validation should be performed in the future by evaluating transcription profiles of NAD- and NADP-dependent ME enzyme under these conditions. Development of a strain with inducible expression of ME can serve as another genetic validation of the observations of our model.

The results found here identify the central metabolic patterns and flux diversion nodes and will help to design genetic or physiological improvement strategies that are translatable to other algae strains and plants.

## Data Availability

The datasets presented in this study can be found in online repositories. The names of the repository/repositories and accession number(s) can be found in the article/**Supplementary Material**.
