# Supplementary material for: Unraveling metabolism underpinning biomass composition shift in Scenedesmus obliquus under simulated outdoor conditions using 13C-fluxomics
Source: Front Plant Sci. 2025 Dec 17;16:1637152. doi: 10.3389/fpls.2025.1637152 (PMC12753895; doi:10.3389/fpls.2025.1637152)
Supplement: Supplementary file 1 [file DataSheet1.docx]

**Unraveling metabolism underpinning biomass composition shift in *Scenedesmus obliquus* under simulated outdoor conditions using ^13^C fluxomics**

Deshpande, A.^1^, Cawthon, B. ^†1^, Loob, J.^1^, Van Wychen, S.^2^, and Laurens, L.M.L *^1^

^1^ BioEconomy and Sustainable Transportation Directorate, National Renewable Energy Laboratory, 15013 Denver West Parkway, Golden, CO, USA

^2^Renewable Resources and Enabling Sciences Center, National Renewable Energy Laboratory, 15013 Denver West Parkway, Golden, CO, USA

*author for correspondence ([lieve.laurens@nrel.gov](mailto:lieve.laurens@nrel.gov))

^†^ Present Address: Department of Chemical and Biomolecular Engineering, University of California, Berkeley, California 94720, United States

**Supporting Photos:**

**
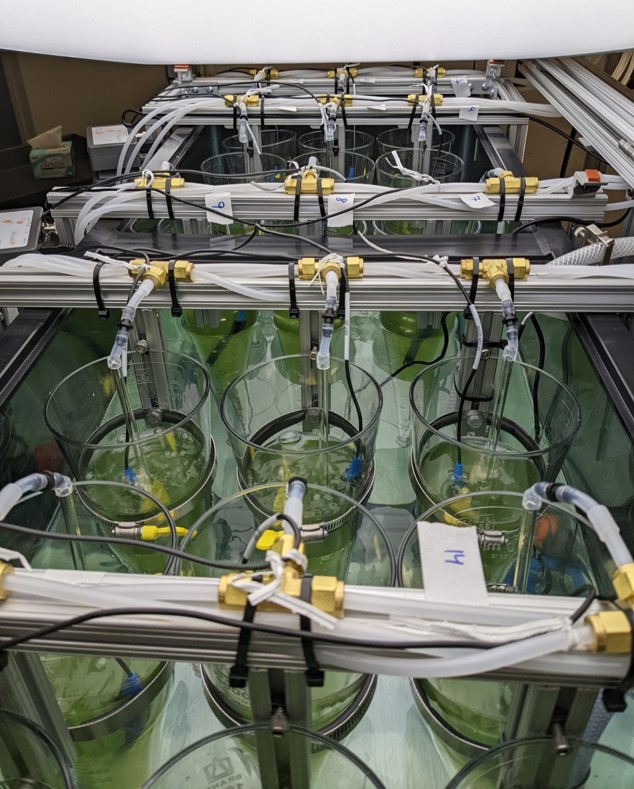
**

Photo S1. A custom-built high throughput 18-position bioreactor, Simulated Algae Growth Environment, SAGE #5. Photo by Mauro Lua and Nick Sweeney.


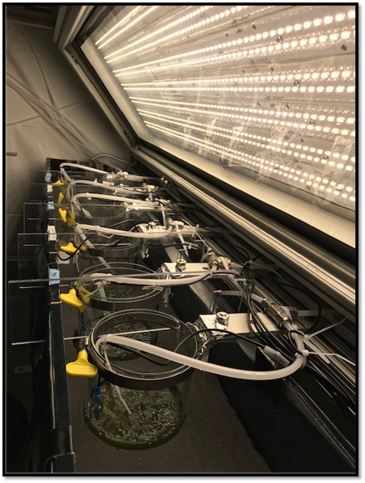


Photo S2. Custom-built 6-position reactor, Simulated Algae Growth Environment, SAGE #3. Reactor is equipped to replicate raceway pond operation with programmable light and temperature, air sparging, and CO_2_ sparging during daytime on feedback pH control. Light is incident from the top once light panel is lowered and reactors are shaded such that light is incident only from the top. Mixing parameters have been fine-tuned mimic raceway pond biomass productivity and composition. This reactor represents an ideal experimental system for performing complex experiments under relevant outdoor conditions. Photo by Mauro Lua and Nick Sweeney.

**Supporting Figures:**


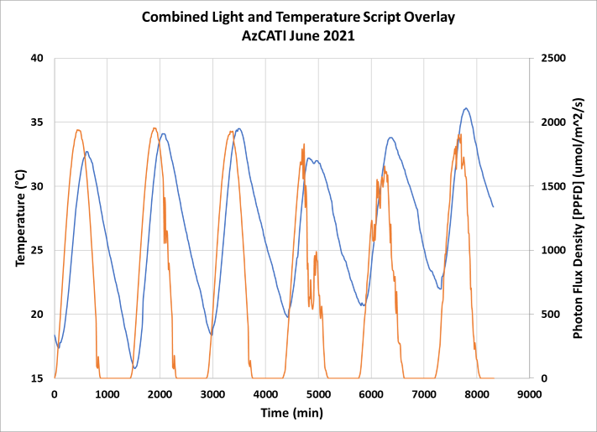


Figure S1. June 2021 light and temperature profile observed at Arizona Centre for Algae Technology and Innovation (AzCATI). For cultivation that exceeded 5 days, upon completion the script was looped.


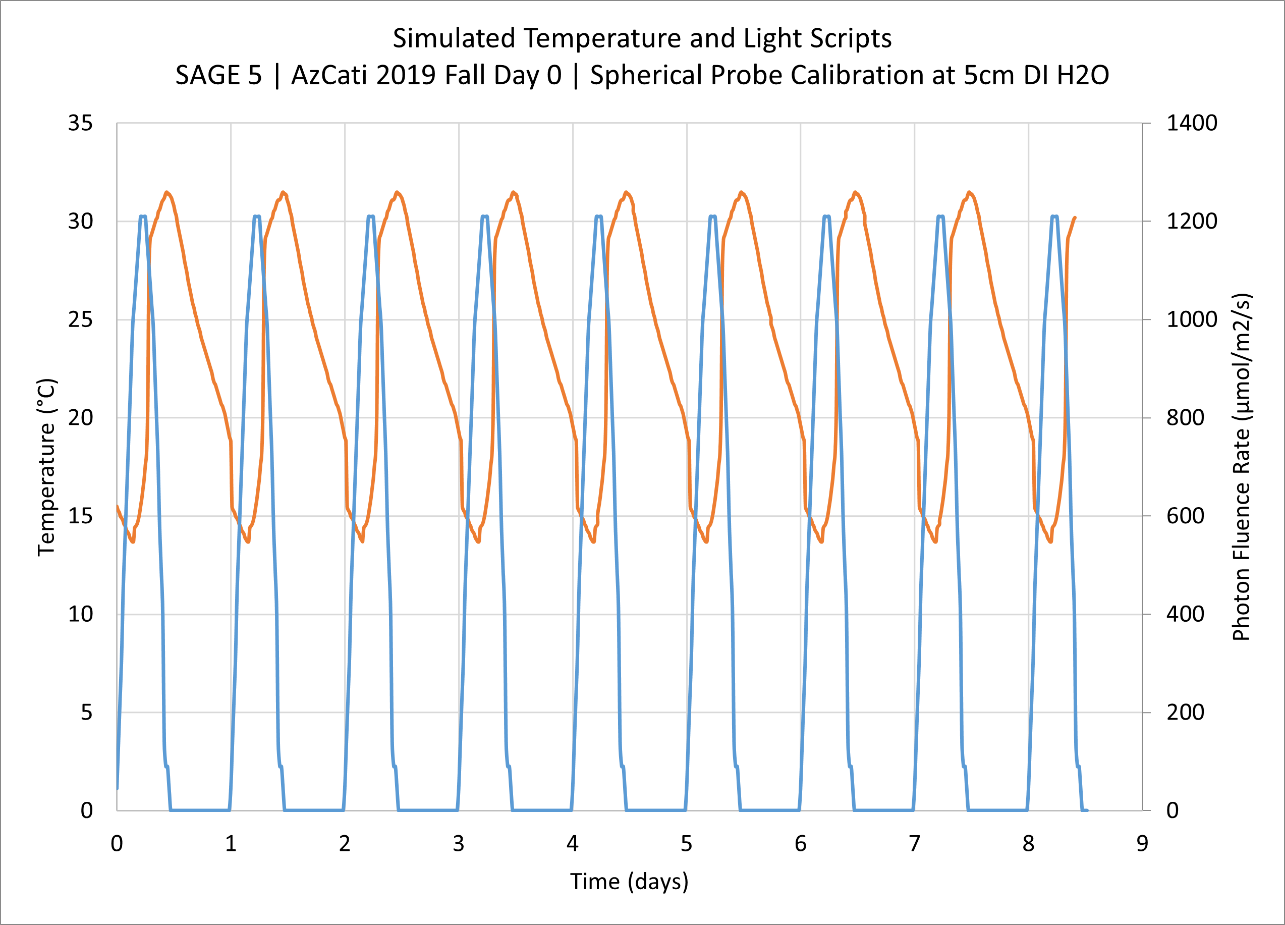


Figure S2. Fall 2019 light and temperature profile observed at Arizona Centre for Algae Technology and Innovation (AzCATI).


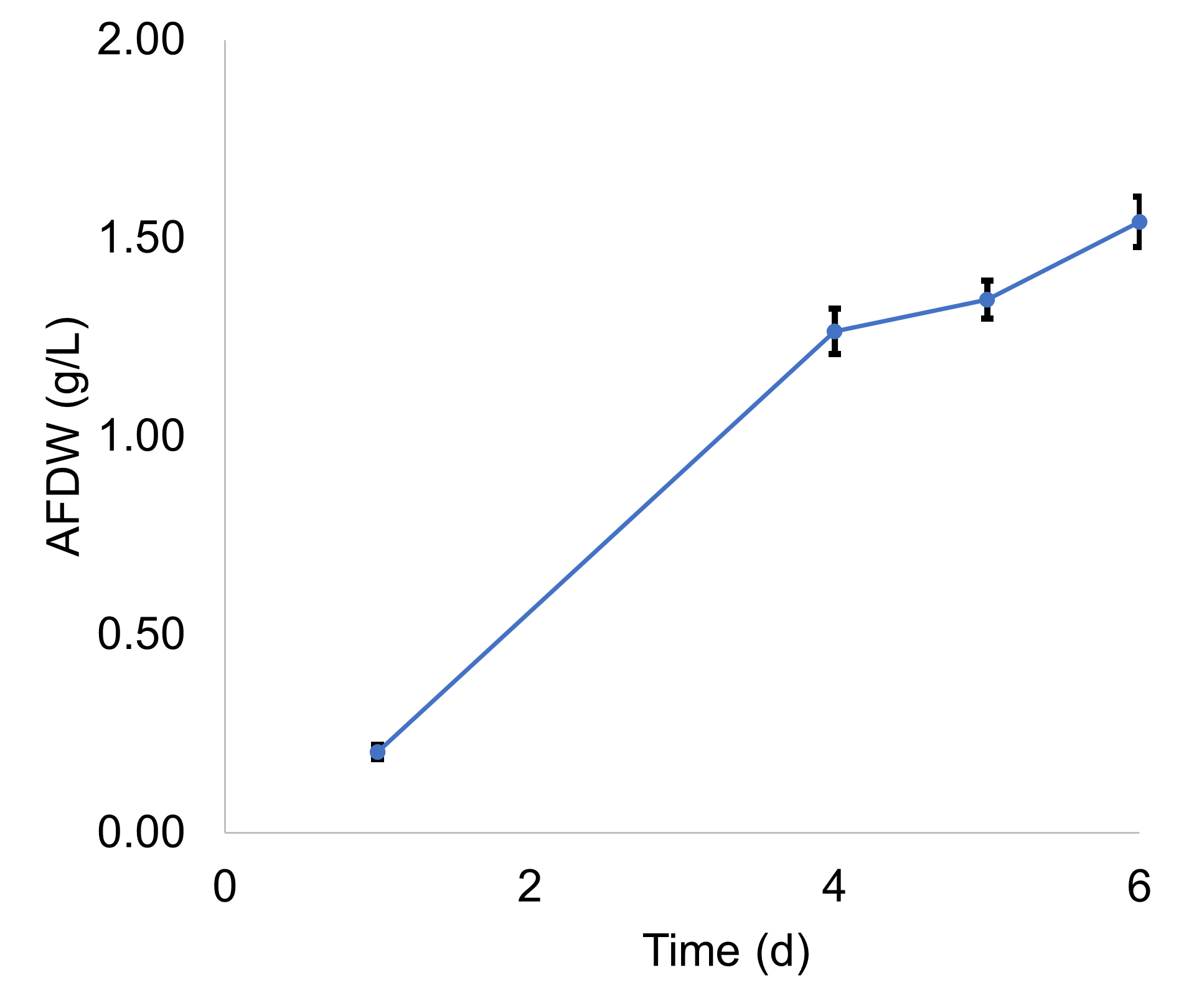


Figure S3. UTEX 393 AFDW data when cultured in SAGE #5 for biomass composition dynamics. Error bars show standard deviation of harvested biological replicates (N=3).


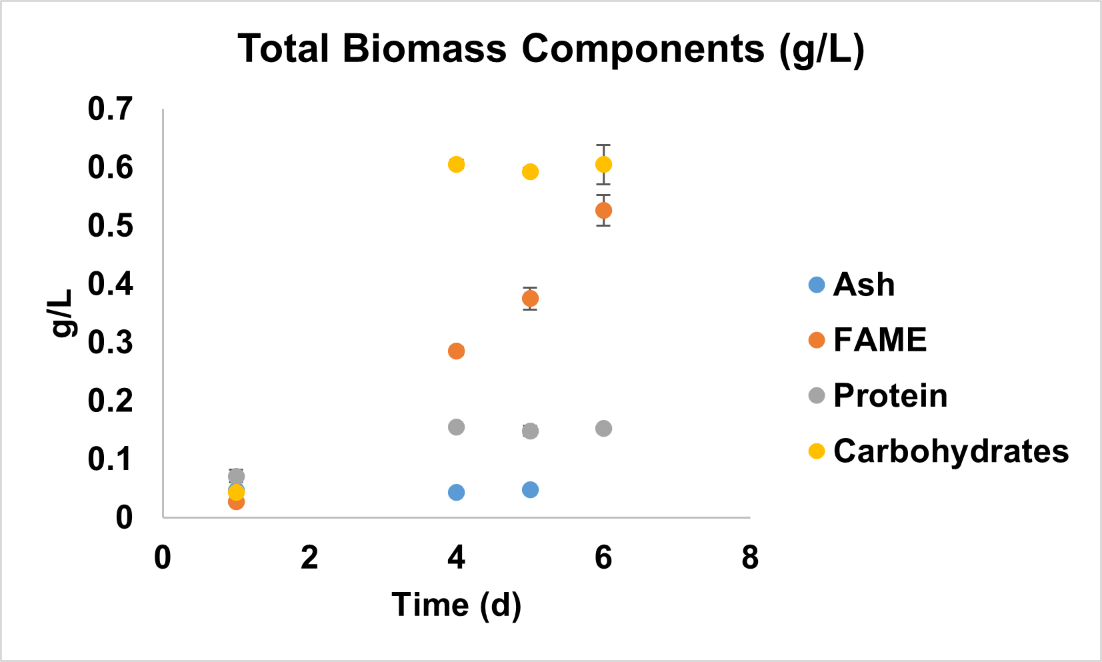


Figure S4. Total mass (g/L) of biomass components over time. Error bars indicate standard deviation of biological replicates (N=3).


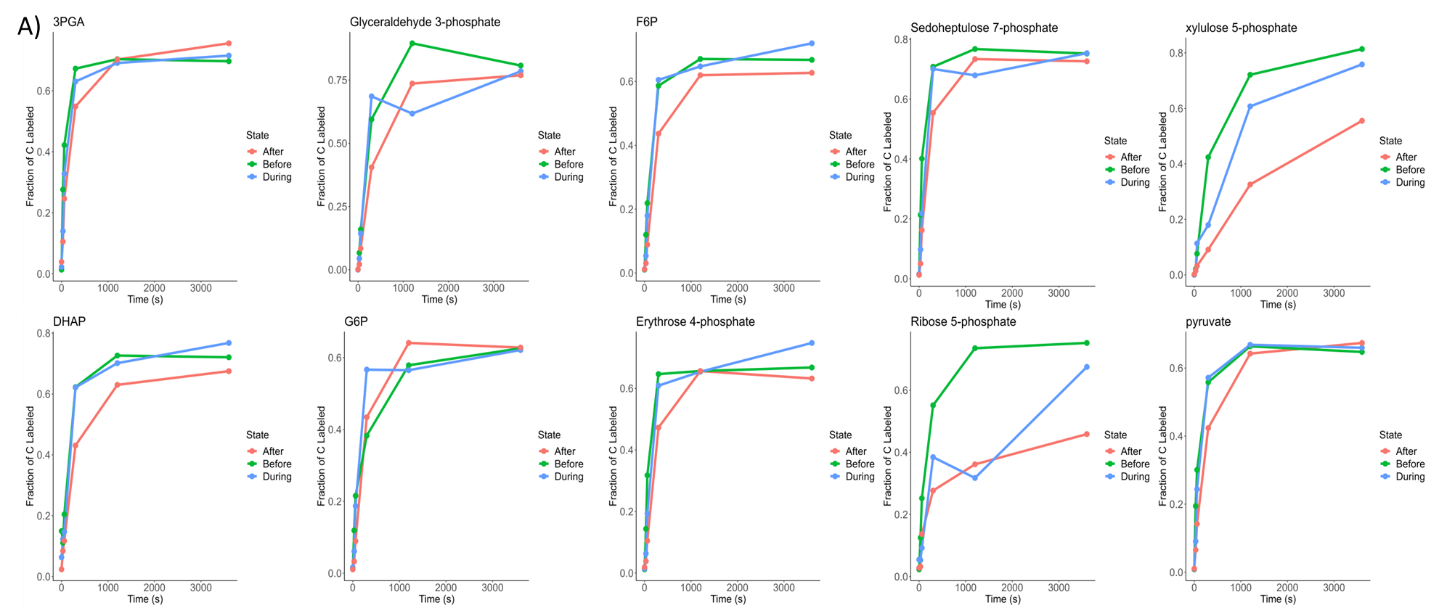


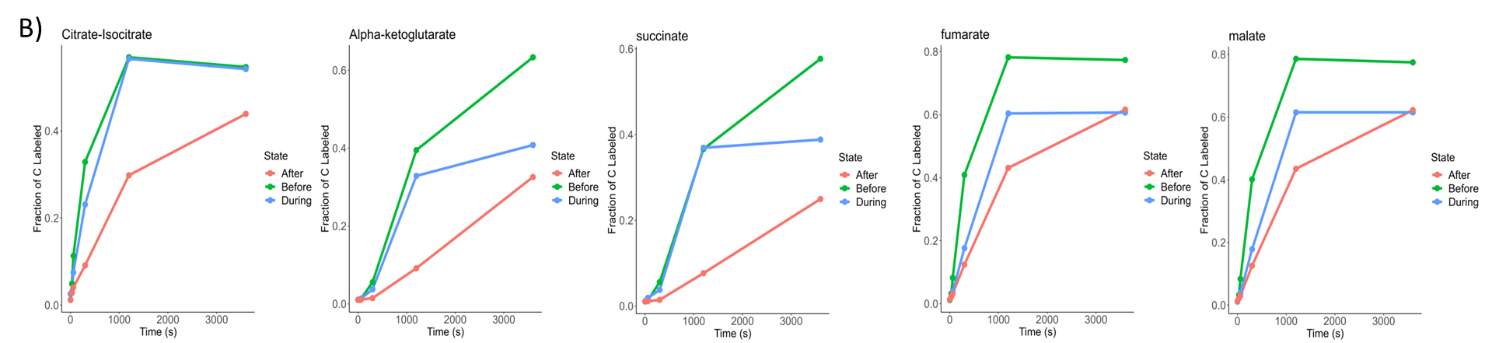


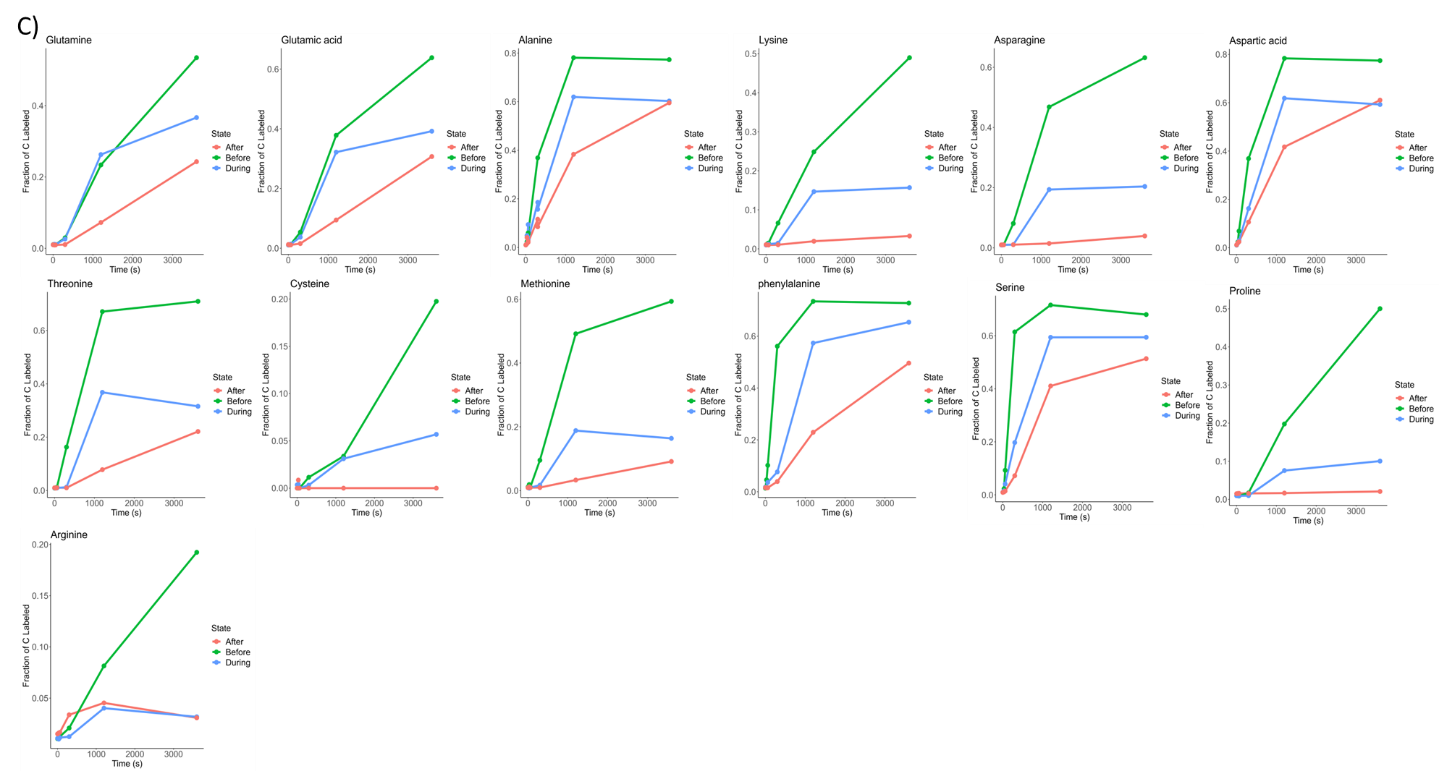


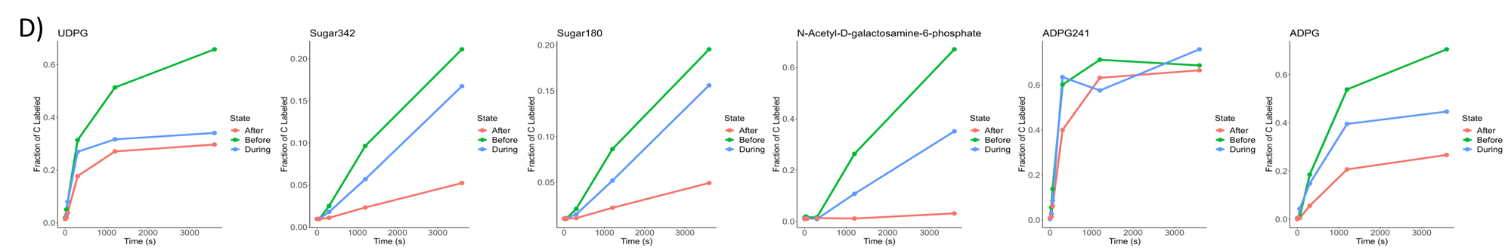


Figure S5. Total ^13^C label incorporation in different metabolites at before, during, and after stages. A) Calvin cycle and Glycolysis, B) TCA cycle, C) Amino acids, D) Other Metabolites. The fraction of C labeled is calculated as $\frac{1}{N}\cdot\sum_{i=1}^{N} M_{i}\cdot i$ where N is the total number of carbons in the metabolite, i is the label, and M_i_ is the fraction of i^th^ isotopologue.


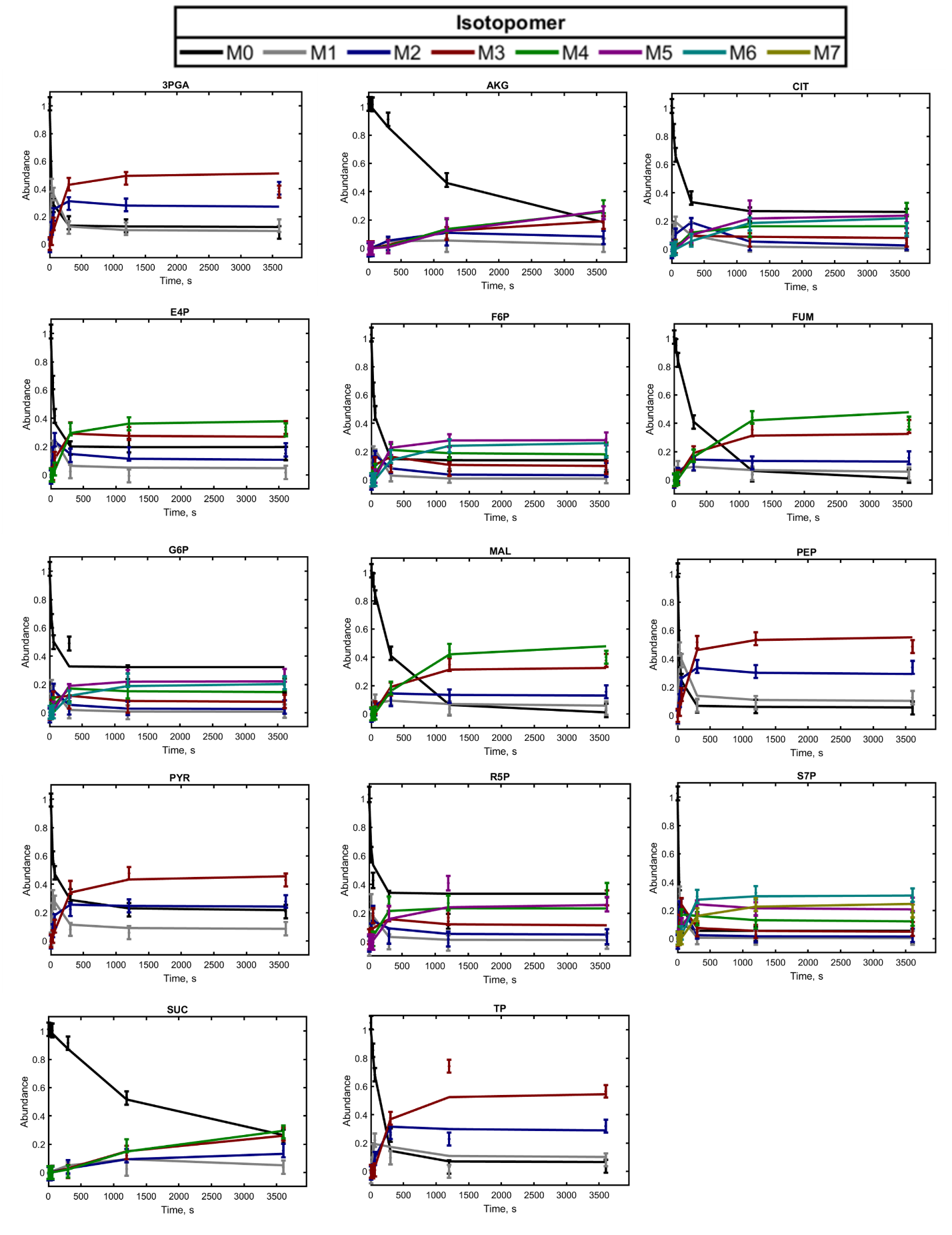


Figure S6. Experimental (dots) and fitted (line) mass isotopomer distribution (MID) “Before” composition shift. Error bars indicate S.D supplied to the INST-MFA model.


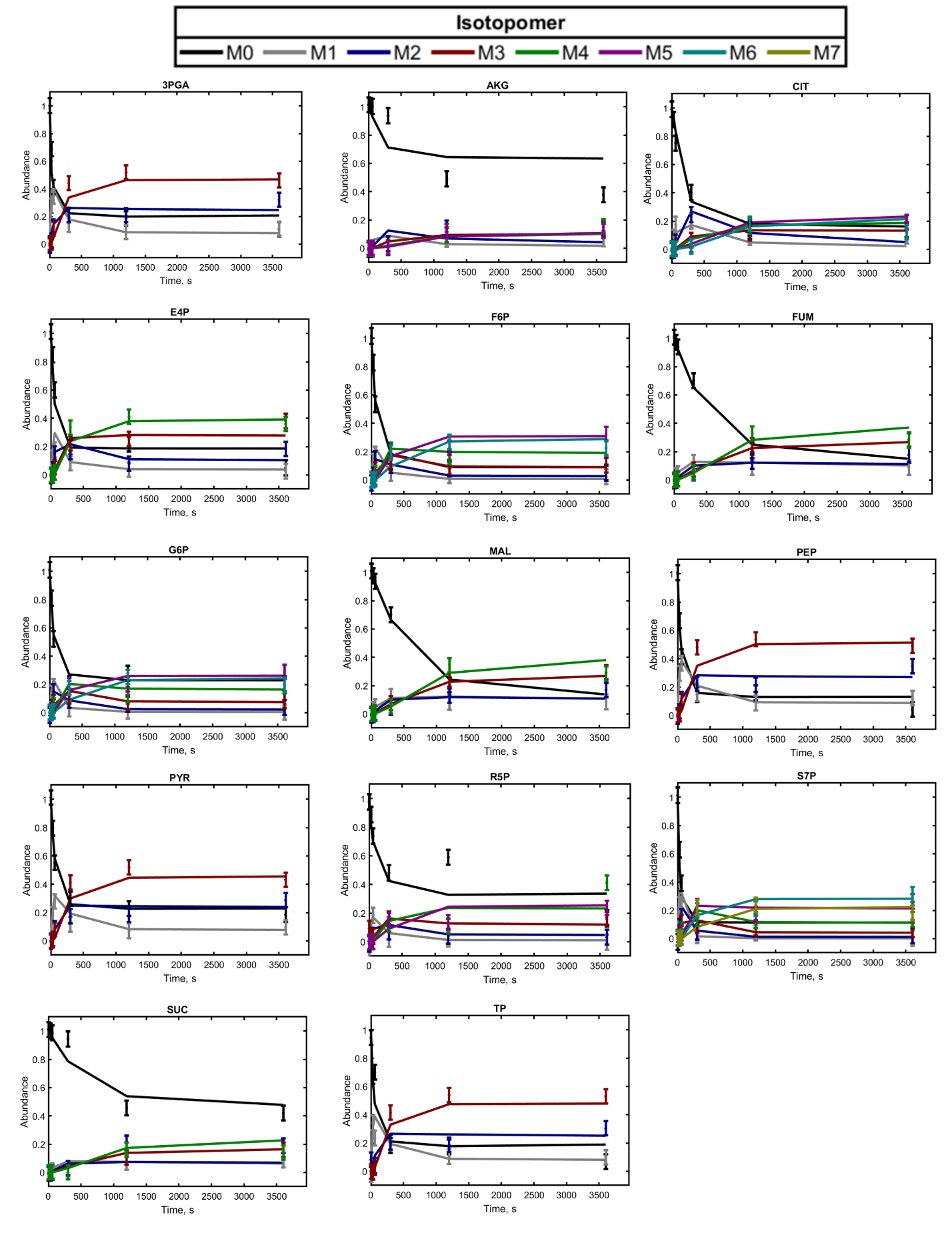


Figure S7. Experimental (dots) and fitted (line) mass isotopomer distribution (MID) “During” composition shift. Error bars indicate S.D supplied to the INST-MFA model.


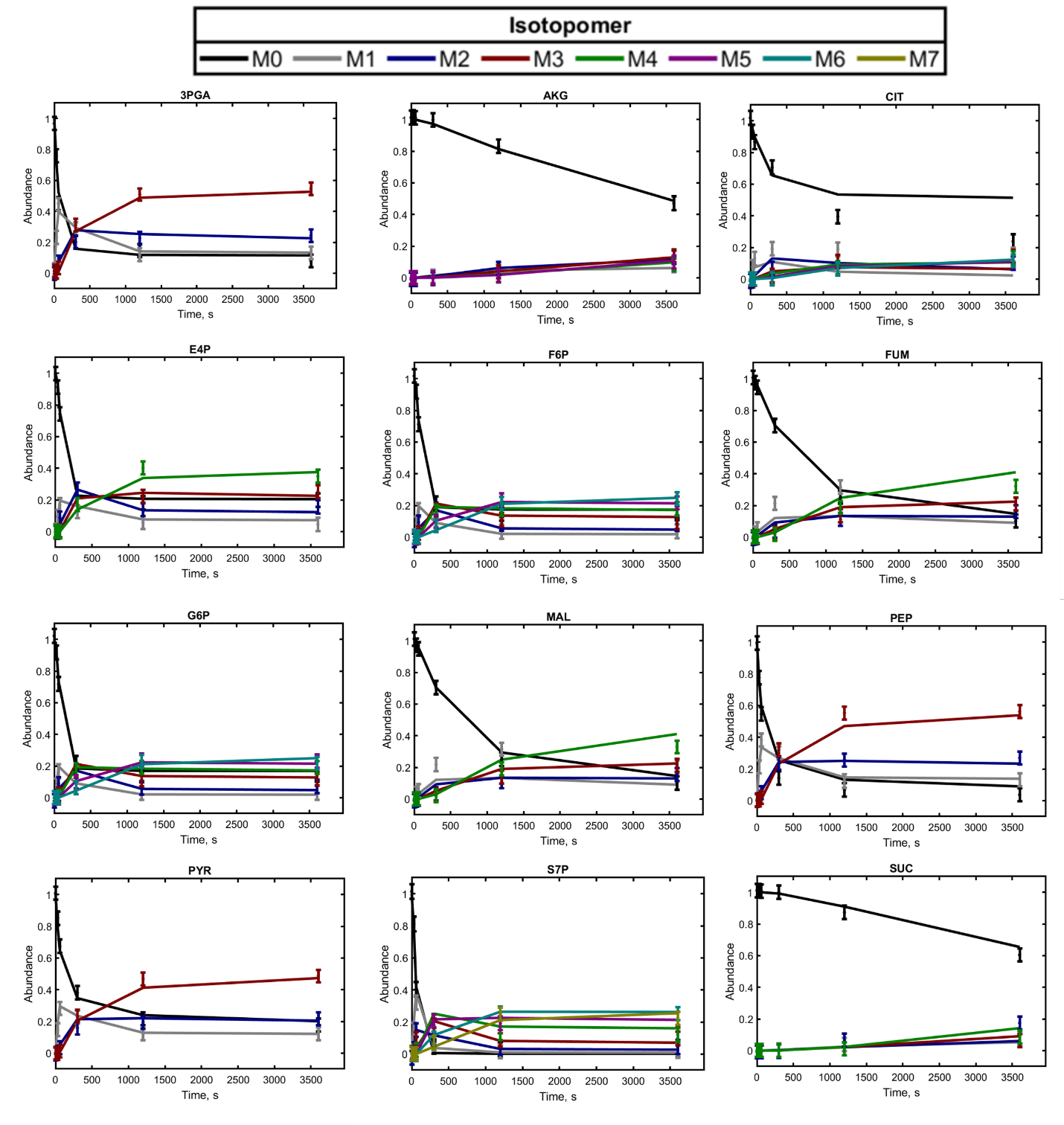


Figure S8. Experimental (dots) and fitted (line) mass isotopomer distribution (MID) “After” composition shift. Error bars indicate S.D supplied to the INST-MFA model.

**Supporting Tables:**

Table S1. Specific growth rate calculated based on OD_750_ measurements 24 hours before sampling for ^13^C labeling. Data represent mean and standard deviation of 6 biological replicates.

| **Stage** | **Specific Growth Rate, µ (h^-1^)** |
| --- | --- |
| Before | 0.0308±0.0003 |
| During | 0.0167±0.0004 |
| After | 0.0016±0.0003 |
